# The Left-Hand 2D:4D Ratio is Superior to the Right-Hand 2D:4D Ratio in Determining the Criminal Potential of Schizophrenia Patients

**DOI:** 10.31083/AP47507

**Published:** 2025-10-20

**Authors:** Elif Emre, Sevler Yıldız, Suna Aydin, Düzgün Şimşek

**Affiliations:** ^1^Department of Anatomy, Firat University, 23119 Elazığ, Turkey; ^2^Department of Psychiatry, Elazığ Fethi Sekin City Hospital, 23280 Elazığ, Turkey; ^3^Department of Cardiovascular Surgery, Elazığ Fethi Sekin City Hospital, 23280 Elazığ, Turkey; ^4^Department of Psychiatry, Elazig Mental Health Hospital, 23200 Elazığ, Turkey

**Keywords:** crime, digit ratios, schizophrenia, 2D:4D

## Abstract

**Background:**

No research has yet examined the potential association between criminal activity, schizophrenia, and the second-to-fourth digit ratio (2D:4D). Therefore, the present study aimed to evaluate if the 2D:4D differs between patients with schizophrenia with and without criminal activities.

**Methods:**

There were 143 male participants in the study: 50 healthy controls and 93 patients with schizophrenia (51 with and 42 without a criminal history). The participants completed the Barratt Impulsiveness Scale (BIS), the Buss–Perry Aggression Questionnaire (BPAQ), and sociodemographic forms. A digital caliper was used to measure finger lengths in order to compute 2D:4D ratios. The Positive and Negative Syndrome Scale (PANSS) was used to measure the severity of schizophrenia.

**Results:**

BPAQ, BIS, and PANSS scores were considerably higher in schizophrenia patients with a criminal background than in those without. Schizophrenia patients with a criminal background had considerably lower right and left 2D:4D ratios than controls. Schizophrenia patients with a criminal background had a significantly lower left 2D:4D ratio than those without. In people with schizophrenia, lower 2D:4D ratios in both hands—particularly the left—are linked to criminal behavior.

**Conclusion:**

The left 2D:4D ratio in a schizophrenia patients with criminal history was a significantly lower compared with those without. Therefore, in individuals with schizophrenia, the left 2D:4D ratio may serve as an early predictor of criminal behavior. This non-invasive anatomical measurement may have the potential to help forensic investigators identify those who are more likely to commit crimes, hence improving public safety.

## Main Points

1. Typical masculine behaviors such as aggression, impulsivity, novelty seeking, and competitiveness are known to be inversely related to the second-to-fourth digit ratio (2D:4D).

2. Individuals who engage in criminal behavior have a lower 2D:4D finger ratio on both hands compared with those with better self-control.

3. As a result, it may be possible to predict an individual’s propensity to commit a crime based on physical characteristics.

4. The left 2D:4D ratio is significantly lower in schizophrenia patients with a criminal history compared with those without.

5. The left-hand 2D:4D ratio is superior to the right-hand 2D:4D ratio in determining the criminal potential of schizophrenia patients.

## 1. Introduction

Over 21 million people worldwide suffer from schizophrenia, a mental illness that impairs cognitive, social, and emotional functioning [1]. Although the pathogenic and psychopathological mechanisms of the disease’s varied nature are strongly supported by research, its etiology is still unclear [2]. The neurodevelopmental hypothesis of schizophrenia suggests that damage to the brain during intrauterine development results in lifelong changes, that manifest as psychosis in early adulthood [3]. The initial symptoms of schizophrenia typically appear shortly after adolescence, with observed gender disparities in the disease process indicating a role for sex hormones in its pathophysiology [4]. Studies have shown that these hormones play a critical role in the development of gray and white matter structures in the brain, as well as in myelination [5, 6]. Since the development of both genitals and digits is regulated by the same *HoxA* and* HoxD* genes, the second-to-fourth digit ratio (2D:4D) serves as an indicator of fetal sex hormone exposure, particularly androgen levels [7, 8]. This 2D:4D ratio remains relatively stable after birth [9]. Therefore, as in many psychiatric disorders, the anatomical 2D:4D finger ratio can be calculated to provide insights into fetal hormonal exposure in schizophrenia patients. Research has been carried out on the 2D:4D in schizophrenia patients, as in many other psychiatric illnesses [10]. The 2D:4D ratio of 843 schizophrenia patients was found to be considerably higher than that of healthy controls in the study by Han *et al*. [11], whereas Venkatasubramanian *et al*. [12] reported a lower 2D:4D ratio in schizophrenia patients compared with controls. Furthermore, Paipa *et al*. [13] demonstrated a correlation between unpleasant and emotional symptoms and the 2D:4D ratio in patients with schizophrenia.

Impulsive and violent actions can be seen in this illness because of the nature of psychotic episodes [14]. Thus, schizophrenic patients who are capable of harming themselves and others are more likely to commit crimes than the general population [15]. When compared with a control group from the general community, 8003 individuals with a diagnosis of schizophrenia were shown to have a significantly higher chance of committing violent crimes, with a predilection for violent criminal offenses [16].

The characteristic masculine tendencies of hostility, novelty seeking, competitiveness, and impulsivity that are frequently observed in schizophrenic patients are inversely proportional to the 2D:4D ratio [17].

Numerous studies have demonstrated that the 2D:4D anatomical finger ratio is linked to a number of disorders [18]. We searched the literature extensively, but were unable to locate any studies that compared the 2D:4D ratio in people with schizophrenia who had criminal histories to the severity of the illness, impulsivity, and aggression. In order to take preventative action and prevent crimes, it may be helpful to identify schizophrenia patients’ propensity for criminal activity early on.

We hypothesize that the 2D:4D finger length ratio, which is assumed to be a likely indicator for some psychiatric disorders, could be different in schizophrenia and might be related with criminal behavior. Therefore, the purpose of this study was to ascertain whether a patient’s propensity to conduct crimes and their 2D:4D ratio are related. It is expected that this non-invasive method will reduce patient comfort and state costs by providing data to both families and law enforcers.

## 2. Materials and Methods

### 2.1 Ethical Considerations

The Ethics Committee of Firat University granted approval for the study (no: 13/2023, date: 27.09.2023). Written consent was obtained from the participants diagnosed with schizophrenia as well as from their legal guardians, adhering to the Helsinki Declaration. The study encompassed 50 healthy male participants with no psychiatric diagnosis, as well as 51 heterosexual male schizophrenia patients involved in crimes admitted to Elazig City Hospital forensic psychiatry service and 42 heterosexual male schizophrenia patients admitted to Elazig Mental Health and Diseases Hospital psychiatry service who were not involved in any crimes between October 1, 2023 and November 30, 2023. Based on the criteria outlined in the 5th edition of the Diagnostic and Statistical Manual of Mental Disorders (DSM-5) published by the American Psychiatric Association, a senior psychiatrist evaluated the patients and diagnosed schizophrenia [19].

Being under the age of 18 years, having any congenital abnormality affecting the upper extremities, having deformities of the hands or fingers, and having major organic disorders that impact skin tone were among the grounds for exclusion. Furthermore, the study included healthy individuals without a history of neurological or psychiatric illnesses, either current or past. Healthy control participants were likewise subject to the exclusion criteria for people with schizophrenia.

### 2.2 Study Scales

The data collection forms used in this study were completed by the participants and the psychiatrist. The interviews lasted approximately 30 minutes for each participant. Written informed consent was obtained from volunteers or legal guardians of persons with schizophrenia before this study began. The Sociodemographic and Clinical Data Form were completed with a psychiatrist’s assistance. This form is a semi-structured form that includes sociodemographic information such as age, marital status, place of residence, education level, occupation, and clinical data such as disease and treatment duration [20]. The Barratt Impulsiveness Scale Version 11 (BIS-11) [21] and Buss–Perry Aggression Questionnaire (BPAQ) [22] were completed by all participants, while the Positive and Negative Syndrome Scale (PANSS) [23] was administered to individuals diagnosed with schizophrenia. Validity and reliability studies of these questionnaires have been conducted in our country and the Turkish translated versions were used.

The scales were used to assess the participants’ impulsivity, aggression traits, and positive-negative syndrome findings of schizophrenia patients in order to determine the link between these psychological traits, criminal potential, and finger ratios.

### 2.3 Barratt Impulsiveness Scale Version 11

This tool is a gold-standard measure that has been essential in shaping current theories of impulse control and has played a key role in studies of impulsivity and its biological, psychological, and behavioral correlates [21]. Güleç *et al*. (2008) [24] adapted the scale into Turkish and performed a validity and reliability evaluation. There are three sub-factors in the scale; attentional impulsiveness, motor impulsiveness, and non-planning impulsiveness.

### 2.4 Buss–Perry Aggression Questionnaire 

The 29-statement BPAQ self-report scale was created to gauge an individual’s level of aggression. It evaluates aggression using the sub-dimensions of hostility, rage, verbal aggression, and physical aggression [22]. Madran and associates (2012) [25] adapted the questionnaire to Turkish.

### 2.5 Positive and Negative Syndrome Scale

The PANSS developed by Kay and colleagues [23] is a comprehensive tool used to assess the severity of positive and negative symptoms, as well as general psychopathology, in individuals with schizophrenia or other psychotic disorders. The scale comprises 30 items, with seven items allocated to positive syndrome symptoms (e.g., delusions, hallucinations, unusual conduct, disorganized thought), seven items to negative syndrome symptoms (e.g., attention problems, anhedonia, avolition, alogia, affective blinking), and sixteen items to general psychopathology (e.g., anxiety, tension, depression, motor retardation) [23]. The Turkish validity and reliability of PANSS were established by Kostakoğlu and colleagues [26], making it a reliable instrument for clinical and research use in Turkish-speaking populations.

### 2.6 Second-to-Fourth Digit Ratio Measurement

According to the previously explained and demonstrated procedure, participants were instructed to hold their hands palmar surface up, dorsal surface flat, and in contact with a hard surface in order to measure finger lengths. The palmar surface was used for measurements. The distance between the fingertip and the middle of the proximal line that separates the finger from the palm [18] was measured. Two independent assessors, who were not aware of the subjects’ group, measured each subject’s index and ring fingers three times. The measures’ arithmetic averages were then determined. The final result was obtained by dividing the length of the second finger by the length of the fourth finger. All measurements were taken using a standard digital caliper that was calibrated to 0.01 mm in order to guarantee accuracy.

### 2.7 Statistical Analysis

**Fig. 1.**
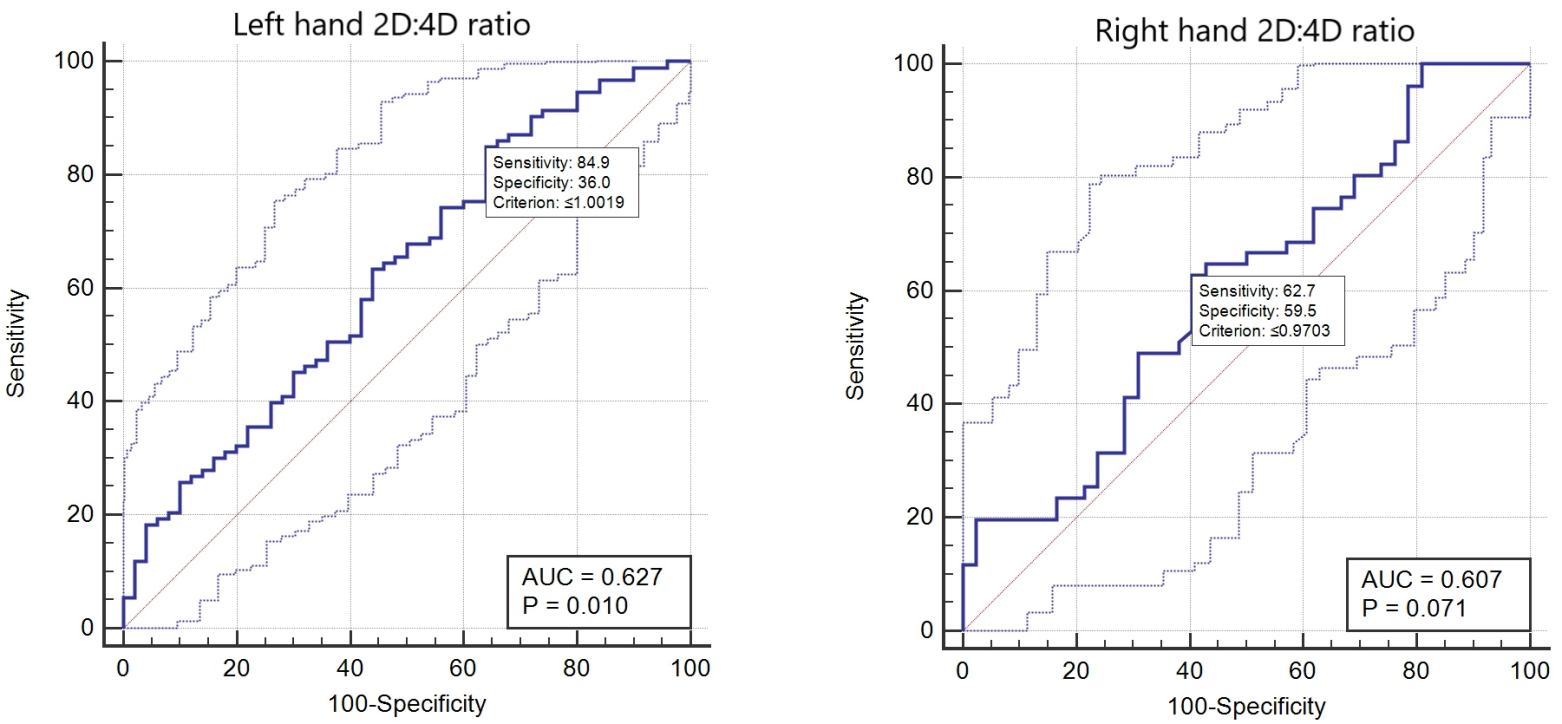
**Right and left-hand 2D:4D sensitivity and specificity of schizophrenia patients who committed crimes**. AUC, area under curve; 2D:4D, the second-to-fourth digit ratio.

Statistical analysis was conducted using BM SPSS Statistics Version 22.0 statistical software package (IBM Corp. Released 2013. IBM SPSS Statistics for Windows, Armonk, NY, USA). The continuous variables’ distributions were evaluated for normality using a combination of histograms and the Kolmogorov-Smirnov test, while the categorical variables were described using frequencies and percentages. The normally distributed numerical parameters were compared using the Student’s *t*-test or one-way analysis of variance (ANOVA) in groups. When numerical parameters did not exhibit a normal distribution, the Mann-Whitney U test or the Kruskal-Wallis test were used for analysis. When appropriate, the Fisher’s Exact or Chi-squared tests were used to compare categorical variables. To determine whether groups differed from one another, we used the Dunn-Bonferroni test for the Kruskal-Wallis test and the Tukey or Bonferroni post hoc tests for the ANOVA. Pearson correlation coefficients or Spearman correlation coefficients were used to assess the degree of correlation between two variables. *p*-values less than 0.05 were regarded as statistically significant. Backward stepwise multiple logistic regression analysis was carried out. To evaluate the model’s fit, Hosmer-Lemeshow goodness-of-fit statistics were used. The following factors were entered into the multiple logistic regression analysis: marital status, alcohol consumption, age, suicide attempt (y/n), duration of illness, and PANSS score. For every predictor, odds ratios (ORs) and 95% confidence intervals (CIs) were computed. The accuracy of the 2D:4D ratio to predict committed crimes in schizophrenia patients was evaluated by receiver operating characteristic (ROC) analysis. The accuracy of the tests was measured by the area under the ROC curve. An area under curve (AUC) close to 1 represents a perfect diagnostic test, whereas an area of 0.5 represents a worthless test. Using Youden’s J statistic, the cut-off value for the 2D:4D ratio to predict crimes committed by patients with schizophrenia were established (Fig. 1).

## 3. Results 

Compared with those who had committed crimes (mean 34 years) and the control group (mean 33 years), the average age of schizophrenia patients who had not committed a crime was greater (mean 42 years). Both the control and patient groups had comparable levels of chronic disease and alcohol use. Alcohol intake was considerably greater among criminal offenders (*p* = 0.034), despite the fact that the prevalence of chronic illness and the usage of antipsychotic medications were identical between schizophrenia patients who committed crimes and those who did not.

Suicide attempts were more common in schizophrenia patients with criminal offenses than in controls and those without criminal offenses (*p* = 0.001 and *p* = 0.034, respectively). Comprehensive results regarding the clinical and sociodemographic characteristics of the patients are shown in Table 1.

**Table 1.** **Comparison of the demographic traits of individuals with schizophrenia who have committed crimes and those who have not**.

		Controls (n = 50)^c^	Committed crimes (n = 51)^cc^	Not committed Crimes (n = 42)^nc^	*p*
Age (y), median (min–max)		33 (22–54)	34 (19–77)	42 (19–77)	c-cc: 0.598
					c-nc: 0.004
					cc-nc: 0.015
Marital Status					c-cc: =0.002
	Single, n (%)	19 (38)	35 (68.6)	39 (92.9)	c-nc: <0.001
	Married, n (%)	31 (62)	16 (31.4)	3 (7.1)	cc-nc: 0.004
Education					
	Illiterate, n (%)	0 (0)	8 (15.7)	8 (19)	c-cc: <0.001
	Primary school, n (%)	0 (0)	26 (51.0)	24 (57.1)	c-nc: <0.001
	High school, n (%)	16 (32)	8 (15.7)	8 (19)	cc-nc: 0.296
	University, n (%)	34 (68)	9 (17.6)	2 (4.8)	–
Place of residence					c-cc: <0.001
	Rural area, n (%)	0 (0)	16 (31.4)	17 (40.5)	c-nc: <0.001
	Urban area, n (%)	50 (100)	35 (68.6)	25 (59.5)	cc-nc: 0.361
Working status					c-cc: <0.001
	Unemployed, n (%)	0 (0)	38 (74.5)	33 (78.6)	c-nc: <0.001
	Employed, n (%)	50 (100)	13 (25.5)	9 (21.4)	cc-nc: 0.646
Other chronic illness					c-cc: 0.028
	Yes, n (%)	2 (4)	9 (17.6)	4 (9.5)	c-nc: 0.285
	No, n (%)	48 (96)	42 (82.4)	38 (90.3)	cc-nc: 0.261
Smoking					c-cc: <0.001
	Yes	19 (38)	45 (88.2)	36 (85.7)	c-nc: <0.001
	No	31 (62)	6 (11.8)	6 (14.3)	cc-nc: 0.718
Alcohol consumption					c-cc: 0.295
	No	44 (88)	41 (80.4)	40 (95.2)	c-nc: 0.220
	Yes	6 (12)	10 (19.6)	2 (4.8)	cc-nc: 0.034
Antipsychotic drug use					c-cc: <0.001
	No	50 (100)	5 (9.8)	2 (4.8)	c-nc: <0.001
	Yes	0 (0)	46 (90.2)	40 (95.2)	cc-nc: 0.359
Suicide attempt					c-cc: =0.001
	No, n (%)	50 (100)	41 (80.4)	40 (95.2)	c-nc: =0.206
	Yes, n (%)	0 (0)	10 (19.6)	2 (4.8)	cc-nc: 0.034
Duration of illness, years, median (min–max)		–	10 (1–30)	12 (1–33)	cc-nc: 0.007
PANSS median (min–max)		–	89 (63–148)	66 (48–125)	cc-nc: 0.001
BPAQ (Mean ± SD)		53.84 ± 12.50	79.12 ± 14.81	70 ± 14.29	c-cc: <0.001
					c-nc: <0.001
					cc-nc: 0.003
BIS-11 (Mean ± SD)		48.94 ± 11.80	76.82 ± 13.64	63.76 ± 13.20	c-cc: <0.001
					c-nc: <0.001
					cc-nc: <0.001

c, control; s, schizophrenia patients; cc, committed crimes; nc, not committed crimes; PANSS, Positive and Negative Syndrome Scale; BPAQ, Buss–Perry Aggression Questionnaire; BIS-11, Barratt Impulsiveness Scale. Categorical variables are presented as n (%); normally distributed variables are presented as mean ± standard deviation while skewed distributed variables are presented as median (min–max); min, minimum; max, maximum; SD, standard deviation.

Compared with the control group (*p* < 0.001, *p* < 0.001) and the group of schizophrenia patients who did not commit crimes (*p* = 0.003, *p* < 0.001), the group of patients who committed crimes scored significantly higher on the BPAQ and BIS-11. The PANSS score was considerably greater in schizophrenia patients who committed crimes than in those who did not (*p* < 0.001) (Table 1).

The right 2D:4D ratios were significantly lower than those of the control group (*p* = 0.004) when all schizophrenia patients were assessed without making a distinction between criminal acts (Table 2). The sensitivity was 62.7 and the specificity of the right hand in criminal discrimination was 59.5 when assessed using the ROC curve (cut-off value ≤0.9703) (Fig. 1). There were statistically significant differences (*p* = 0.002 and *p* = 0.014, respectively) between the control group and the right and left-hand 2D:4D ratios of schizophrenia patients who committed crimes. There was no discernible difference between the schizophrenic patients who did not commit crimes and the control group. Between schizophrenia patients who committed crimes and those who did not, there was a significant difference in the left-hand 2D:4D ratio (*p* = 0.017) (Table 2). The sensitivity was 84.9 and the specificity was 36.0 according to the ROC curve analysis (cut-off value ≤1.0019) (Fig. 1). There was a positive link between the right and left 2D:4D ratios, but not between the patients’ age, scale scores, or duration of illness (Table 3).

**Table 2.** **Right and left-hand 2D:4D ratios in control and schizophrenia patients groups**.

	Control (n = 50)^c^	Schizophrenia Patients (n = 93)		*p*
Right 2D:4D ratio, n ± SD	0.992 ± 0.034	0.976 ± 0.032^s^		c-s: 0.004
		Committed crimes (n = 51)^cc^	Not committed crimes (n = 42)^nc^	c-cc: 0.002
				c-nc: 0.323
		0.969 ± 0.028	0.982 ± 0.034	cc-nc: 0.142
Left 2D:4D ratio, n ± SD	0.988 ± 0.034	0.976 ± 0.042^s^		c-s: 0.092
		Committed crimes (n = 51)^cc^	Not committed crimes (n = 42)^nc^	c-cc: 0.014
				c-nc: 0.998
		0.966 ± 0.035	0.988 ± 0.046	cc-nc: 0.017

c, control; s, schizophrenia patients; cc, committed crimes; nc, not committed crimes. *p*: *p* values in each group after post-hoc test, normally distributed variables are presented as mean ± standard deviation.

**Table 3.** **Correlation between age, length of illness, and other factors and the 2D:4D ratio of the right and left hands of people with schizophrenia who have committed crimes and those who have not**.

		All Patients (n = 93)		Committed Crimes (n = 51)		Not Committed Crimes (n = 42)	
		Right 2D:4D Ratio	Left 2D:4D Ratio	Right 2D:4D Ratio	Left 2D:4D Ratio	Right 2D:4D Ratio	Left 2D:4D Ratio
Right 2D:4D ratio	*r*	–	0.613	–	0.694	–	0.633
	*p*	–	<0.001	–	<0.001	–	<0.001
Left 2D:4D ratio	*r*	0.613	–	0.694	–	0.633	–
	*p*	<0.001	–	<0.001	–	<0.001	–
Age	*r*	0.030	–0.038	0.020	–0.017	0.171	0.090
	*p*	0.718	0.654	0.888	0.907	0.279	0.571
BPAQ	*r*	–0.037	–0.055	0.253	0.126	0.163	0.040
	*p*	0.661	0.517	0.073	0.378	0.303	0.800
BIS-11	*r*	–0.126	–0.163	–0.069	–0.066	0.227	0.079
	*p*	0.135	0.051	0.631	0.643	0.149	0.621
Duration of illness	*r*	0.089	0.008	–0.017	–0.082	0.162	0.067
	*p*	0.395	0.939	0.904	0.569	0.307	0.671
PANSS	*r*	–0.048	–0.085	0.120	0.068	–0.036	0.100
	*p*	0.647	0.420	0.403	0.637	0.822	0.527

A low left 2D:4D ratio (OR = 0.53, 95% CI: 0.21–0.76; *p *= 0.047), married status rather than single status (OR = 8.94, 95% CI: 1.98–40.41; *p *= 0.004), a high PANSS score (OR = 1.04, 95% CI: 1.01–1.08; *p *= 0.021), and a high BIS-11 score (OR = 1.06, 95% CI: 1.01–1.11; *p *= 0.018) were all linked to crime in individuals with schizophrenia, according to data from the logistic regression analysis (Table 4).

**Table 4.** **Independent indicators of criminal behavior in individuals with schizophrenia**.

	Adjusted	
Risk factor	OR (95% CI)	*p*
Marital status (being married vs single)	8.94 (1.98–40.41)	0.004
PANSS	1.04 (1.01–1.08)	0.021
BIS-11	1.06 (1.01–1.11)	0.018
Left 2D:4D ratio	0.53 (0.21–0.76)	0.047

OR, odds ratio; 95% CI, 95% confidence interval; the *p*-value of the Hosmer-Lemeshow test was 0.351.

## 4. Discussion

Those with schizophrenia who committed crimes showed significantly lower left-hand 2D:4D ratios than those without criminal records. A low 2D:4D ratio in men has been strongly linked in the past to disinhibition, thrill seeking, and risk-taking behaviors [27]. In our analysis, we also found that patients with schizophrenia exhibited a low left-hand 2D:4D ratio, which was linked to their risk-taking and thrill-seeking tendencies. A previous study that included women found that people with schizophrenia had low 2D:4D ratios [12]. These results were in line with those that had been previously published [14, 27, 28].

For the first time, the present study found a correlation between criminality and the 2D:4D ratio in individuals with schizophrenia. High levels of impulsivity and violence have been previously linked to schizophrenia, which raises the risk of criminal behavior [14, 29]. People with schizophrenia are four to seven times more likely to commit violent crimes such as murder and assault, and they are also four to six times more likely to engage in general aggressive behaviors including verbal and physical threats, according to some previous research [30, 31]. In 2023, Gao *et al*. [32]. found a direct correlation between aggression and schizophrenia in a study of 367 patients with schizophrenia.

By assessing the degree of impulsivity and aggression as well as the 2D:4D ratio, we showed in this study that people with schizophrenia who have committed crimes have high levels of impulsivity and aggression, and that their left-hand 2D:4D ratio is low. Furthermore, the 2D:4D ratio in schizophrenia has been the subject of multiple reports in the last years [33]. Venkatasubramanian *et al.* [12] discovered that patients with schizophrenia had a higher finger ratio than healthy controls, while Han *et al*. [11] demonstrated that patients with schizophrenia had a low 2D:4D ratio.

As a result, there is still disagreement over the right-left 2D:4D ratio in schizophrenia patients. In our study, the specificity of the left-hand ratio was 36.0 with a sensitivity of 84.9 when schizophrenia patients who committed crimes were assessed using the ROC curve (cut-off value ≤1.0019). In contrast, the specificity of the right hand for crime discrimination was 62.7 with a sensitivity of 59.5 when only the right hand cut-off value ≤0.9703 was accepted in schizophrenia patients with a criminal background. The specificity and sensitivity of the 2D:4D right and left-hand ratios in connection with criminal offense involvement were compared for the first time in this study.

Another finding of this study was that people with schizophrenia who commit crimes tend to be younger than those who do not. Young individuals are susceptible to schizophrenia, and the more severe the illness is, the more abilities are lost [34, 35].

Nonetheless, it is well known that criminal activity peaks in the twenties and then declines [36]. Similarly, previous research has shown that violence is higher in younger individuals with schizophrenia. Thus, it can be concluded that young people with schizophrenia are more likely to commit crimes [33]. We believe that the age categories of offenders and non-offenders in our study did not have an impact on the outcomes of our 2D:4D ratio analyses. This is because the 2D:4D, or the length ratio of the index finger to the ring finger, is a biological marker that remains constant throughout life [9, 37].

The results of this study’s regression analysis show that impulsivity, marital status, severity of symptoms, and a low left-handed 2D:4D ratio may all make people with schizophrenia more likely to commit crimes. This is because Gurkan and colleagues’ [38] similar investigation revealed that individuals with schizophrenia who had committed crimes had higher aggressiveness scores among those with the same marital status and PANSS scores.

According to this study, schizophrenia patients who have committed crimes frequently attempt suicide, and this is linked to a low left-hand 2D:4D ratio. According to Lenz and colleagues [39], men who committed suicide had a lower 2D:4D ratio than controls.

Consequently, these results align with our findings. According to a different study, impulsive people may be more likely to commit suicide [40].

Some limitations of the study are the limited sample size and the exclusive male composition of our sample group. Also, another drawback is the excessively poor specificity, which suggests a large probability of false positives. This may be somewhat preventing the research’s clinical application.

Due to these challenges, we were unable to evaluate our results in light of the specifics of the crimes. Nevertheless, we anticipate that finger length measurement, an inexpensive, non-invasive, and widely accessible technique, might be used as a tool in predicting the likelihood of criminal behavior despite these limitations.

## 5. Conclusion

Patients with schizophrenia who have committed crimes had decreased 2D:4D finger ratios on both hands. Compared with schizophrenia individuals who have not committed a crime, this decline was more pronounced in the left hand. One should remember that this prediction tool is adequately trustworthy; however, more research is needed to determine its limitations and usability in clinical practice. Consequently, it is possible to anticipate in advance the likelihood of criminal activity by taking into account these facts. This low-cost, non-invasive technique can help identify individuals with schizophrenia more easily and identify those who are at risk of committing crimes before they do.

## Availability of Data and Materials

The datasets generated during and analyzed during the current study are available from the corresponding author on reasonable request.
